# Establishing a High-Quality Congenital Cardiac Surgery Program in a Developing Country: Lessons Learned

**DOI:** 10.3389/fped.2020.00357

**Published:** 2020-07-30

**Authors:** Issam El Rassi, Jana Assy, Mariam Arabi, Marianne Nimah Majdalani, Khalid Yunis, Rana Sharara, Marie Maroun-Aouad, Roland Khaddoum, Sahar Siddik-Sayyid, Carine Foz, Ziad Bulbul, Fadi Bitar

**Affiliations:** ^1^Department of Surgery, The American University of Beirut-Medical Center, Beirut, Lebanon; ^2^Department of Pediatrics and Adolescent Medicine, The American University of Beirut-Medical Center, Beirut, Lebanon; ^3^Department of Anesthesiology at the Children's Heart Center, The American University of Beirut-Medical Center, Beirut, Lebanon

**Keywords:** cardiac surgery, developing countries, children, congenital heart disease, pediatric cardiac program

## Abstract

**Background:** Developing countries are profoundly affected by the burden of congenital heart disease (CHD) because of limited resources, poverty, cost, and inefficient governance. The outcome of pediatric cardiac surgery in developing countries is suboptimal, and the availability of sustainable programs is minimal.

**Aim:** This study describes the establishment of a high quality *in-situ* pediatric cardiac surgery program in Lebanon, a limited resource country.

**Methods:** We enrolled all patients operated for CHD at the Children's Heart Center at the American University of Beirut between January 2014 and December 2018. Financial information was obtained. We established a partnership between the state, private University hospital, and philanthropic organizations to support the program.

**Results:** In 5 years, 856 consecutive patients underwent 993 surgical procedures. Neonates and infants constituted 22.5 and 22.6% of our cohort, respectively. Most patients (82.6%) underwent one cardiac procedure. Our results were similar to those of the Society of Thoracic Surgeons (STS) harvest and to the expected mortalities in RACHS-1 scores with an overall mortality of 2.8%. The government (Public) covered 43% of the hospital bill, the Philanthropic organizations covered 30%, and the Private hospital provided a 25% discount. The parents' out-of-pocket contribution included another 2%. The average cost per patient, including neonates, was $19,800.

**Conclusion:** High standard pediatric cardiac surgery programs can be achieved in limited-resource countries, with outcome measures comparable to developed countries. We established a viable financial model through a tripartite partnership between Public, Private, and Philanthropy (3P system) to provide high caliber care to children with CHD.

## Introduction

The need for quality congenital heart surgery in developing countries is growing (1–3), and children with cardiovascular disease have always been, and still are severely underserved (1). Major congenital anomalies are responsible for ~7% of all neonatal deaths in developing countries, among which 25% are due to congenital heart disease alone (2–4).

Because of resource needs, cost, and affordability, congenital heart disease creates an enormous burden in low- and middle-income countries. Most reports coming from the third world, describe the negative aspects of congenital heart surgery, with challenges unfamiliar to the developed world. The service is rarely a vital government priority; it is often just an appendage to an adult service (2), and the results and outcomes are inferior to those of developed countries (5–7). There are even negative comments concerning patient management, with cost considerations leading to deviation from standard practice guidelines (8, 9).

There is a wide array of definitions for “developing countries.” For this reason, the World Bank is shifting toward using the income-based classification, although it also includes a wide range of different economies classified in the same group (10, 11). This heterogeneity may explain why despite discouraging reports, some upper-middle-income countries in Eastern Europe, Asia, and the Middle East, have defeated the “curse” of developing countries and built successful congenital cardiac surgery programs with overall mortalities around 5% (12–16). However, sustaining these programs is a real challenge.

Lebanon, a small Middle Eastern country with a 6 million population, is classified as a developing middle-income country. We established an *in-situ* pediatric cardiac surgery program at the American University of Beirut with outcome measures comparable to those of North American and West European centers, and with overall mortality below 3%. This study presents the results and the elements of success and sustainability of the program.

## Methods

Between January 2014 and December 2018, we enrolled all patients operated for congenital heart disease at the Children's Heart Center at the American University of Beirut in Lebanon in this study. The only excluded patients were premature neonates, on mechanical ventilation, operated for the closure of a patent ductus arteriosus. We excluded procedures for re-operations for bleeding, wound revision for infection, and epicardial pacemaker insertion.

Following Institutional Review Board approval, the following data were documented: demographic information, surgical procedure, complexity class, mortality and mortality, infection, length of stay in the intensive care unit (ICU), and the total length of stay in the hospital. We obtained financial information from the billing department for all patients operated during the last year of the study (2018). Finances included the total amount of each bill, the extent of government coverage, humanitarian assistance, out-of-pocket contribution, and hospital discount.

We analyzed the patients according to cardiac defects and surgical procedures. When multiple defects were diagnosed, the anomaly was labeled according to the main defect. When various defects were repaired during the same operation, the procedure was labeled according to the main defect repaired, or the higher surgical complexity class. For example, a transposition with VSD was labeled as transposition repair; a VSD with an ASD was labeled as VSD repair. The same patient with a tetralogy of Fallot may have been operated for the insertion of a Blalock–Taussig shunt, and then for tetralogy repair. The surgical procedures were distributed according to complexity, using the RACHS-1 complexity categories (17) (Risk Adjustment for Congenital Heart Surgery). We used the 5-year harvest (2014–2018) from the STS (6) (Society of Thoracic Surgeons) to benchmark outcomes. Data are presented as numbers, percentages, and mean with standard deviation. We performed tabulations and calculations using Microsoft Excel 2016.

## Results

### Demographics and Distribution

In 5 years, 856 consecutive patients underwent 993 surgical procedures. Table 1 shows the patients' characteristics. The mean age was 3.64 ± 5.3 years, and 57% were males. Most patients (82.6%) underwent only one cardiac procedure, while 149 patients underwent multiple surgeries. Most re-operations (82%) were the classically staged surgeries needed for the repair of the initial cardiac anomaly, and 14% were due to surgical complications, imperfect repairs, or residual defects. Four percent were cardiac procedures performed for the repair of abnormalities unrelated to the initial malformation or one of its consequences (Table 1).

**Table 1:** Patients' characteristics and postoperative course.

Total number of patients	856	ICU length of stay	5.7 ± 6.2 days
Total number of procedures	993	Range	1–30 days
Male sex	57%	Total hospital stay	12.2 ± 8.8 days
		range	1–69 days
**Procedure/Patient**			
-1 Procedure	707 (82.6%)	Infection	12.4%
-2 Procedures	128 (15.0%)	- Pneumonia	5.9%
-3 Procedures	16 (1.8%)	-Urinary tract	1.9%
-4 Procedures	5 (0.6%)	-Superficial wound	1.9%
		-Deep wound	1.3%
-Redo procedures	286 (29%)	-Blood stream	1.1%
-Staged redo surgery	234 (82%)	-Endocarditis	0.3%
-Surgery for complication	40 (14%)		
-Unrelated redo surgery	12 (4%)	Peritoneal dialysis	5.3%
		Neurologic complications	2.1%

*ICU, Intensive care unit*.

Age groups are listed in **Table 3** and compared to the abundant 5-year STS harvest; neonates and infants accounted for 22.5 and 22.6%, respectively, whereas children constituted 50%, and adults 4.9%.

### Anomalies and Procedures

Table 2 lists the different cardiac anomalies and procedures. The most common anomaly was tetralogy of Fallot and its variants including pulmonary atresia with ventricular septal defect (VSD), and absent pulmonary valve syndrome. Tetralogy of Fallot, VSD, and various single ventricle categories constituted 50% of the patient cohort. Among the 993 cardiac procedures, VSD closure (15.4%) was most commonly performed, followed by simple tetralogy repair (9.1%), then Blalock–Taussig shunt insertion (6.5%). There were 38 different cardiac procedures listed, but the first seven constituted 50% of all interventions. The distribution of procedures into the various complexity categories is shown in Table 3 and was grossly similar to the STS harvest.

**Table 2:** List of all initial cardiac anomalies and all surgical procedures.

**Cardiac anomalies in 856 patients**	**Nb**	**%**	**993 Surgical procedures**	**Nb**	**%**
Tetralogy and variants	162	18.9%	Ventricular septal defect	153	15.4%
-Tetralogy	90		Simple tetralogy repair	90	9.1%
-Pulmonary atresia + VSD	41		Blalock–Taussig shunt	65	6.5%
-Absent valve syndrome	10		Pulmonary valve or conduit insertion	60	6.0%
			Mitral valve surgery	56	5.6%
Ventricular septal defect	153	17.9%	Aortic Coarctation	50	5.0%
Single ventricle	117	13.7%	Rastelli, REV, or Nikaidoh procedure	48	4.8%
Aortic Coarctation/Arch hypoplasia	50	5.8%	Total cavo-pulmonary connection	47	4.7%
Transposition of the great arteries	48	5.6%	Partial cavo-pulmonary connection	46	4.6%
Complete atrioventricular septal defect	48	5.6%	Complete atrioventricular septal defect	44	4.4%
Subaortic membrane	30	3.5%	Arterial switch procedure	40	4.0%
Aortic valve stenosis or regurgitation^#^	30	3.5%	Subaortic membrane resection	30	3.0%
Atrial septal defect closure	26	3.0%	Aortic valve surgery	30	3.0%
Mitral valve stenosis or regurgitation^*^	26	3.0%	Pulmonary artery Banding	28	2.8%
Patent ductus arteriosus closure	23	2.7%	Atrial septal defect	26	2.6%
Double outlet right ventricle	22	2.6%	Patent ductus arteriosus	25	2.5%
Partial anomalous pulmonary veins	19	2.2%	Total anomalous pulmonary veins	21	2.1%
Total anomalous pulmonary veins	18	2.1%	Partial anomalous pulmonary veins	19	1.9%
Partial atrioventricular septal defect	11	1.3%	Unifocalization of pulmonary collaterals	19	1.9%
Truncus arteriosus	11	1.3%	Interrupted aortic arch	13	1.3%
Interrupted aortic arch repair	10	1.2%	Norwood, or comprehensive Norwood	13	1.3%
Hypertrophic obstructive cardiomyopathy	8	0.9%	Hybrid procedure for HLHS	12	1.2%
Pulmonary atresia with intact septum	7	0.8%	Partial atrioventricular septal defect	11	1.1%
Pulmonary valve stenosis	6	0.7%	Tricuspid valve surgery	9	0.9%
Ebstein disease	6	0.7%	Subaortic myectomy ± Mitral procedure	8	0.8%
Shone syndrome	4	0.5%	Pulmonary stenosis		
Aortic ring	4	0.5%			
			Double chamber right ventricle		
Isolated tricuspid valve regurgitation			Heart transplantation		
ALCAPA			Right ventricle false aneurysm		
Aortic arch hypoplasia			Aortic ring		
Supra-valvar aortic stenosis			ALCAPA		
Pulmonary artery sling	17 patients 2%		Aortic arch hypoplasia	30 patients 3%	
Cortriatriatum			Supra-valvar aortic stenosis		
Anomalous coronary artery			Pulmonary artery sling		
Iatrogenic right atrial perforation			Cortriatriatum		
Right atrial thrombosis			Anomalous coronary artery		
			Right atrial perforation repair		
			Right atrial thrombus removal		
			Damus–Kay–Stansel procedure		

# *Due to Endocarditis in two patients*.

* *Due to Endocarditis in one patient*.

*ALCAPA, Anomalous left coronary artery from the pulmonary artery*.

**Table 3:** Patient numbers and mortality according to age and complexity.

	**AUBMC 2014–2018 cohort**		**STS 2014–2018 Harvest**	
	**Number of procedures %**	**Mortality %**	**Number of procedures %**	**Mortality %**
**Overall**	993	2.81	122,439	2.90
**Age groups**				
- Neonates (0–28 days)	223 (22.5)	5.82	29,076 (23.7)	8.20
- Infants (28 days−1 year)	224 (22.6)	2.23	40,753 (33.3)	2.90
- Children (1–18 years)	497 (50.0)	2.01	42,593 (34.8)	1
- Adults (>18 years)	49 (4.9)	0	10,017 (8.2)	1.20
**Complexity**	**Number of procedures %**	**Mortality %**	**Expected percentage %**	**Expected mortality %**
1	92 (9.2)	0	12	0.6
2	382 (38.4)	1.57	36	1.6
RACHS-1–3	383 (38.6)	3.39	36	3.1
Categories 4	108 (10.9)	7.4	12	7.7
5 + 6	28 (2.8)	3.5	4.2	11.2–13.1

*AUBMC, American University of Beirut Medical Center; RACHS-1, Risk adjusted classification for congenital heart surgery; STS, Society of Thoracic Surgeons*.

### Complications and Mortality

The postoperative course of the patients is presented in Table 1. The mean length of stay in ICU was 5.7 days (± 6.2 days), and the total hospital stay was 12.2 days (±8.8 days). Infection was reported in 12.4% of the patients. The most common infection was pneumonia (5.9%), followed by superficial (1.9%) or deep wound infection (1.3%). We used near-infrared spectroscopy (NIRS) monitoring in many of our patients to measure cerebral oxygenation (rSO2). We observed acute clinical neurologic complications in 2.1% of the cases, 30% of whom died. Most neurologic complications (80%) were seizures in neonates with complex anomalies. Peritoneal dialysis for acute kidney injury was initiated in 5.3% of the patients, 22% of whom died.

Mortality according to age and complexity is shown in Table 3. It is compared to the STS harvest and the expected mortality of the RACHS-1 score. Our overall mortality was 2.8%. The overall mortality, as well as mortality in most age groups, was slightly better in our patient cohort than in the STS harvest, except for patients between 1 and 18 years of age, where our mortality reached 2.01% compared to 1% for the STS. According to complexity categories, our mortality was remarkably similar to the expected mortalities in most of the RACHS-1 scores. In the most complex categories (RACHS 5 and 6), one of the 28 patients of our series died (3.5%), whereas the expected mortality ranges between 11 and 13%.

### Financial Implications

**Figure 1:**
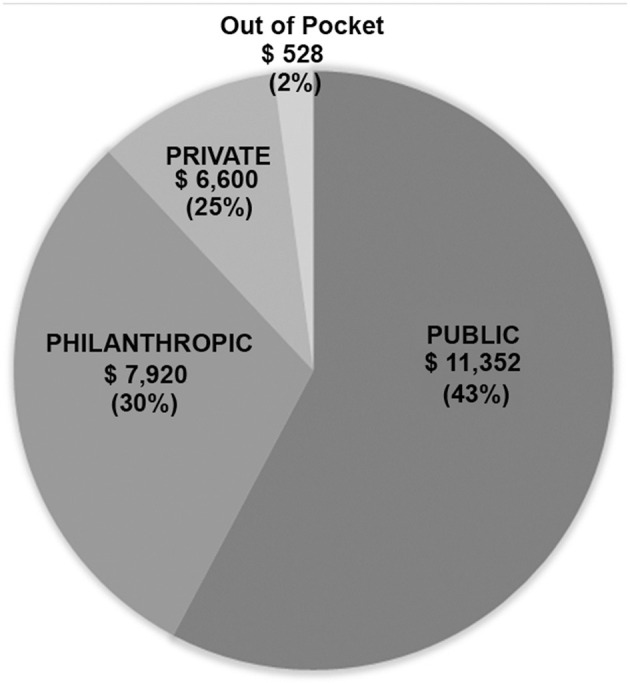
3P Partnership: Pie chart showing distribution of financial coverage between the three partners.

Finances were obtained for all patients operated during the last year of the study (2018) and involved 159 patients. Data are shown in Table 4 and Figure 1. The total billed amount was $4,363,000 with $27,450 (±$13,685) being the average. Private patients, self-covered or covered by insurance, constituted only 11.3% of the group, whereas 141/159 (88.7%) were state-covered patients. The bills of state covered patients averaged $26,400 ± $14,542 per patient. The state covered 43% on average of each bill ($11,352 ± $9,542). The parents' out-of-pocket contribution covered another 2% ($528 per patient). The humanitarian organizations covered $7,920 per patient, which constituted 30% of the bills, and the hospital waived and absorbed the remaining 25% ($6,600). The average cost per state covered patient, including hospital discount, was $19,600, including neonatal surgeries.

**Table 4:** Cost and financial coverage for year 2018 (159 patients).

	**Number of patients**	**Total billed**	**Per patient (average)**
**Total**	159	4,363,000$	27,450$ (±14.950$) (Range: 11.500$-121.500$)
**Private patients**	18 (11.3%)		
Insurance	12	463,000$	38,500$
Self-payers	6	178,000$	29,600$
**State covered patients**	141 (88.7%)	3,722,000$	26,400$
State coverage (Public)	141	2,148,500$	11,352$ (43%)
Humanitarian help (Philanthropic)	141	1,126,650$	7,920$ (30%)
Out of pocket contribution (Private)	24	79,550$	528$ (2%)
Hospital contribution (Private)	141	367,300$	6,600$ (25%)

## Discussion

Developing countries are profoundly affected by the burden of congenital heart disease (CHD) because of poverty, limited resources, cost, absence of dedicated children's hospitals, and low patient volume in some areas (2, 5). Besides, congenital anomalies are more prevalent in countries with high fertility rates, where the age structure of the population shows higher proportions of younger people and a disproportionate number of children born with CHD (18). Adult wage-earning patients are usually able to pay for a part of health care, compared to young couples at the beginning of their professional lives (1, 16). Even in high-income countries, parents are often financially affected by a child born with CHD. A study conducted in the United States showed that the financial impact on families was felt almost immediately following the diagnosis of the child's heart disease (19). Despite being insured, all families identified many out-of-pocket costs during their child's hospitalization, with many citing food and lodging as consuming most of their resources. Mothers quit or lose their jobs for the inability to place their children in daycare.

A significant obstacle in the way of achieving sustainable programs in limited-resource countries is resource distribution. Health expenditure is a large part of the Gross Domestic Product (GDP) in developed nations, but only a small fraction in low and middle-income countries (20). Hence, children with CHD are not a priority for the government when more prevalent diseases and other social problems are not entirely under control. Also, inefficient governance, lack of auditing, and corruption prevent the fair distribution of the resources when they exist, and more money is spent on servicing debt and equipping the armed forces, than on health and education combined (20, 21).

Nevertheless, cardiac surgery for CHD remains an essential part of pediatric health care for the following considerations: (1) our profession's moral imperative is to treat these children, regardless of any financial concern; (2) denying care for children with CHD, would not improve the care of other diseases: due to inefficient governance, denying funds to CHD doesn't mean that these funds would be allocated to strengthen other health care issues; (3) according to the United Nations, treating CHD offers considerable economic value in terms of human development (22, 23). In 2018, Cardarelli et al. analyzed 446 patients operated in several low and middle-income countries to estimate the improvement in the United Nations Human Development Indicators for each survivor as a proxy for the long-term benefits of the intervention. He concluded that treating these children was equivalent to 1,484 years of schooling, and 67,640,000$ gained by the entire cohort (22).

Financial issues are one of the most critical problems facing high-quality care. The key to any financially viable business, including healthcare, is to avoid a negative balance. Thus, the cost of surgery must be reimbursed to achieve a sustainable system. Everyone agrees that some kind of partnership between the several stakeholders is necessary to accomplish this goal (1, 2, 21). With limited state health insurance in most developing countries, philanthropic organizations, local, or international, have partnered with local governments to cater to most of the intermittent cardiac surgical care in extremely impoverished regions of the globe (2, 5, 8). However, for regular, sustainable, thus more expensive surgical care, more partners are needed. Therefore, private healthcare institutions including universities, as well as families who can afford to pay part of the cost, shall be included in what we have called the “3P” system which entails a partnership between The Public (state/government), the Private (private hospitals; families, insurance), and the Philanthropy organizations.

With the increasing cost of cardiac surgery, governmental insurance covers in Lebanon only 43% of what congenital cardiac surgery costs in a tertiary care private University hospital like ours, the American University of Beirut Medical Center (AUBMC). Only a small minority of families can afford to pay the difference or can provide expensive private insurance to cover the rest. Hence, our university hospital, as a private, not-for-profit hospital, grants a 25% reduction on all bills when a philanthropic organization is engaged. The charitable organizations are the loyal partner of both the government and the hospital, to make sure that no patient would be denied cardiac surgery for lack of funds. The cost of cardiac surgery is thus efficiently covered according to the scheme in Figure 1. All patients presenting to our institution are admitted and operated under this scheme, including neonates, and patients with high complexity anomalies without any discrimination.

Because of highly variable annual clinical volumes, political instability, and lack of financial resources, low and middle-income countries are not usually attractive for private specialized congenital cardiac centers (16, 23); most pediatric cardiac centers are thus located in public hospitals, or not-for-profit private healthcare institutions often supported by NGOs (23). The long-term stability of these cardiac centers is entirely related to the support received from local political authority and/or university (20). Universities have proven to be an essential ingredient for success, and congenital heart surgery is best based in a university hospital. The university ensures transparency, accountability, and avoidance of the governmental bureaucracy (17, 20). Also, the availability of other subspecialties helps to improve outcomes further, and peer pressure and academic values dictate strategies, rigor, and adherence to guidelines. Our hospital, AUBMC, follows the North American standards of care, and has earned international accreditations attesting to its standards in patient-centered care, nursing, and graduate medical education. We believe like many others, that highly specialized centers, similar to those present in developed countries, can be established in developing countries, and are the best way to obtain similar outcomes (21, 24–26). For this reason, and from the outset, the “Children's Heart Center” was modeled on examples from existing programs in Western Europe and North America, and has been equipped to provide for all what is needed for state-of-the-art congenital heart surgery, including temporary and permanent mechanical cardiopulmonary support, modern catheterization facilities, echocardiography machines with transesophageal probes, continuous renal replacement therapy, and incubators for neonates and premature babies. Trained key medical and paramedical staff is also believed to be an essential element for success and lack of trained personnel has been recognized as a major limiting factor in emerging nations (7, 8). Our center was initially established by the recruitment of credentialed and well-trained physicians in the United States and Europe with their commitment to jump-start the program. The establishment of educational and research services are essential components for the sustainability of the program. In addition, our high caliber fellowship training programs graduate future physicians. Some of our graduates seek additional training abroad and later return to join our team, thus providing the needed human resources to sustain the program. In addition, many of our trainees move to build and support other programs in the region.

The congenital practice, including the Intensive Care Unit (ICU), is totally independent, geographically and financially, from the adult cardiac surgical unit; they only share two dedicated operating rooms and the operating room personnel with assigned time slots for each surgeon.

Nearly 90% of children with heart disease in low- and middle-income countries do not have access to treatment (18). Most NGOs (71%) helping these children are based outside the regions they serve and provide intermittent help for a small number of children. Fifty-one percent of the NGOs perform five missions per year, and 20% accomplish one mission per year (3, 9). The 3P system adopted in our setting allows NGOs to deliver impactful and sustainable care to all patients presenting with congenital heart disease, in collaboration with a high quality *in-situ* cardiac center.

The average cost per patient, including hospital discount, is $19,800 in our setting, whereas, in many developing countries, the reported cost of pediatric cardiac surgery is described within a range of $3.000–10.000 (22, 27). This significant difference is due to the strategic decision taken by our program to model our pediatric cardiac surgery center using examples from the developed world, which incurs substantial costs related to maintenance, equipment, safety, training, excellent outcome measures, and appropriate salaries to retain the talented human resources. The price is also higher than in other developing countries due to the absence of patient selection and the inclusion of complex neonatal and infant surgery in our program (about 45% of our patients are neonates and infants). However, this cost remains far from the standard surgical charges at centers in the western world, although the outcomes are comparable. The 3P system described above has been sustainable because the philanthropic partner proved to be a robust partner, covering a significant portion (30%) of the cost. The state, as a public partner (covering 43% of the bill), is the most reliable partner. The private partner (the hospital, the families) is the most fragile; the hospital provides a 25% discount to all humanitarian cases but relies heavily on its two partners to sustain its ability to provide the needed expenses to maintain the high standards of care. The Hospital operates at cost to support this strategy, as long as the cost is covered by the 3P partnership, and the system doesn't function at a loss.

Successful outcomes have been reported by teams operating in low-resource countries; however, this was true only when surgeons shied away from performing neonatal, adult congenital, and complex surgeries (7, 13). Cost considerations can also push surgeons away from practice guidelines, resorting to less expensive palliative surgeries, with less stormy postoperative courses, which may cause clinical harm in the long run, and ultimately, additional financial losses (8, 9, 13). We have learned this by observing the experience of other centers, and occasionally by managing several patients treated initially in different local and regional institutions.

Also, several factors specific to developing countries complicate congenital cardiac surgery (7): A significant number of patients, especially among the refugee population in our country, suffer from malnutrition and late diagnosis, creating unusual weight distribution, and complexity categories unique to developing countries. Because of delayed diagnosis, the percentage of our infant group was less than the STS harvest (22 vs. 33%) and significantly higher in the children group (50 vs. 35%; Table 3). Late diagnosis, with the resulting recurrent infections, pulmonary hypertension, and malnutrition may also explain the slightly increased mortality and morbidity among our children group compared to the STS harvest (2.1 vs. 1%), and also the slight increase in the length of stay (28).

Dedicated pediatric cardiac intensive care units are still scarce in emerging nations, and most of the programs, are part of an adult cardiac surgical ICU or a general pediatric ICU (7). Dedicated pediatric cardiac ICUs are not yet universally present, even in the developed world. In a survey on 54 pediatric cardiac intensive care programs in the United States and Europe, 35% of the institutions had a dedicated cardiac ICU, whereas, in 65%, pediatric cardiac patients were cared for within a general pediatric cardiac intensive care unit (29). Moreover, recruiting dedicated pediatric cardiac intensivists remains a challenge, and is still considered an expensive luxury in developing countries (30). For the above reasons, we have elected to assign a specific location for cardiac children in a pediatric ICU. This decision took into consideration the annual limited patient volume in our country, and the necessity to concentrate on skilled human resources in one unit. All recruited pediatric intensivists underwent additional fellowship training in pediatric cardiac surgery.

Brain drain remains one of the most serious threats facing the skilled human resources in low-income countries. In addition to being tempted by better remuneration, they have to face the active recruitment efforts deployed by prestigious institutions in other developing nations (2, 20). As a strategic plan to retain its recruits, our hospital guarantees a decent remuneration to crucial medical staff, attractive enough to make the rate of job abandonment very low. Furthermore, salaries of all nursing staff are continuously revised to avoid competitive recruitment by other healthcare institutions.

In small countries with reduced patient numbers, it remains challenging to maintain competency and skills, especially for anomalies requiring complex surgical techniques and dedicated postoperative care. For example, in 1 year, our unit manages <10 patients with transposition of the great arteries, <4 patients needing unifocalization, and performs <2 Norwood procedures. Surprisingly, the impact of low patient numbers was not reflected by higher mortality in the highest complexity classes. This may be explained by the fact that some of our faculty are hired with prior experience obtained abroad.

Our blueprint toward success started with a vision to develop a center that delivers state-of-the-art care with excellence in teaching, education, and research in a compassionate environment. We established a system that pulls together a team that works harmoniously in a multidisciplinary fashion and fosters collaboration among various pediatric disciplines. Our success depended on our university's commitment to supporting the center by providing the needed resources, allocating the facilities, and encouraging its growth and development.

Moreover, the program cultivated a culture of giving to our community. It established partnerships and collaborations with humanitarian organizations that contributed to the establishment of a financially sustainable system.

## Conclusion

High quality pediatric cardiac surgery programs can be achieved in low-resource countries, with results comparable to developed countries. In summary, the elements of success of such program include:

- A properly designed and well-equipped modern facility, preferably in a university hospital setting.
- A well-trained, experienced, and dedicated medical and paramedical personnel.
- A well-planned financial strategy that includes a partnership between a philanthropic organization, a private university hospital, and the public sector which allows the pediatric cardiac program to function without financial loss, thus achieving long term sustainability.

## Data Availability Statement

The datasets generated for this study are available on request to the corresponding author.

## Ethics Statement

The studies involving human participants were reviewed and approved by Human Research Protection Program and Institutional Review Board at the American University of Beirut Medical Center. Written informed consent from the participants' legal guardian/next of kin was not required to participate in this study in accordance with the national legislation and the institutional requirements.

## Author Contributions

IE and FB contributed conception and design of the study, organized the database, performed the statistical analysis, and wrote the first draft of the manuscript. JA, MM, RS, KY, MM-A, RK, SS-S, CF, ZB, and MA organized the database. All authors contributed to manuscript revision, read, and approved the submitted version.

## Conflict of Interest

The authors declare that the research was conducted in the absence of any commercial or financial relationships that could be construed as a potential conflict of interest.
